# XS: a FASTQ read simulator

**DOI:** 10.1186/1756-0500-7-40

**Published:** 2014-01-16

**Authors:** Diogo Pratas, Armando J Pinho, João M O S Rodrigues

**Affiliations:** 1 Signal Processing Lab, IEETA/DETI University of Aveiro, Aveiro 3810–193, Portugal

## Abstract

**Background:**

The emerging next-generation sequencing (NGS) is bringing, besides the natural huge amounts of data, an avalanche of new specialized tools (for analysis, compression, alignment, among others) and large public and private network infrastructures. Therefore, a direct necessity of specific simulation tools for testing and benchmarking is rising, such as a flexible and portable FASTQ read simulator, without the need of a reference sequence, yet correctly prepared for producing approximately the same characteristics as real data.

**Findings:**

We present XS, a skilled FASTQ read simulation tool, flexible, portable (does not need a reference sequence) and tunable in terms of sequence complexity. It has several running modes, depending on the time and memory available, and is aimed at testing computing infrastructures, namely cloud computing of large-scale projects, and testing FASTQ compression algorithms. Moreover, XS offers the possibility of simulating the three main FASTQ components individually (headers, DNA sequences and quality-scores).

**Conclusions:**

XS provides an efficient and convenient method for fast simulation of FASTQ files, such as those from Ion Torrent (currently uncovered by other simulators), Roche-454, Illumina and ABI-SOLiD sequencing machines. This tool is publicly available at http://bioinformatics.ua.pt/software/xs/.

## Background

Large-scale high-profile projects such as the 1000 Genomes Project and The Cancer Genome Atlas, as well as prizes that reward cheaper, faster, less prone to errors and higher-throughput sequencing methodologies favored the emergence of next-generation sequencing (NGS), marking an historical moment for biomedical and social sciences, one that promised to settle long-standing questions and, concomitantly, opened a new set of challenges in individual genomics and personalized medicine [1,2].

Consequently, hardly thinkable situations, such as genomics sequencing projects having a larger fraction of the budget allocated to the storage infrastructures than to the biological part, become a reality. As a way to tackle this problem, several local and distributed data storage infrastructures emerged with techniques associated to lower costs, such as the Amazon Cloud for the 1000 Genomes Project data. Furthermore, concerns derived from information exceeding the growth of the media capacity to store them boosted specialized compression algorithms [3-10].

Since thousands of scientists want to access and analyze NGS data, high-profile projects collaborate with several institutions in different countries to operate distributed computing and data storage infrastructures. On the other hand, there are many private research groups that are creating local infrastructures to store information, in order to increase interactivity and to minimize the analysis time. Moreover, there is a rising problem associated with individual genomics and personalized medicine which is confidentiality. Therefore, only a few human genome sequences will be publicly accessible. Mostly by the mentioned reasons, it is expected that huge private storage infrastructures will be created in the following years.

Private storage infrastructures follow a construction plan that leads to an implementation phase resulting in a testing phase. One of the commonly performed tests is the load test, after slowly downloading data from genome banks publicly available from the Internet (1000 Genomes Project, The Cancer Genome Atlas, among others). Although simulation of data requires computational power, the simulation time of a large file is usually less than the time used to download the same file, mainly because computational processing bandwidth is higher than the Internet’s bandwidth. For instance, assume that a research group wants to download one hundred sequenced genomes in FASTQ format using a perfect 100 Mbits per second download link. Now assume that each genome, with a three-fold average coverage, occupies approximately 25 GB and about 8.4 GB when compressed. They need more than 19 hours to download the compressed version and 57 hours to download the uncompressed version. Moreover, the compressed version requires additional time for decompression. On the other hand, a simulation approach needs less than 1 hour, even without launching several processes at the same time.

FASTQ [11] is the most used NGS file format. The FASTQ file format provides a simple extension to the well known FASTA format, which is the ability to store a numeric quality score associated with each nucleotide in a sequence. Therefore, a FASTQ file consists of three different sub-sources, namely the headers (identifiers), DNA bases and quality scores.

The first DNA sequence simulation tools emerged to test phylogenetic hypotheses [12] and for sequence analysis [13-16]. The second wave of simulators introduced the ability to cope with the DNA structure [17-19], beyond the introduction of specific simulation purposes such as error correcting codes [20], RNA secondary structures [21] and DNA sub-string distributions [22].

With the introduction of the NGS, several simulation tools have been proposed. Richter *et al.* and Balzer *et al.* proposed, respectively, MetaSim [23] and FlowSim [24], aware that the subsequence context influences error rates in Roche-454 and Illumina sequencing [25,26]. MetaSim simulates collections of reads that reflect the diverse taxonomical composition of typical metagenome datasets, based on a database of given genomes. On the other hand, Flowsim simulates advanced error modeling and quality scores, although it is only limited to Roche-454 sequencing. Huang *et al.* proposed ART [27], which simulates sequencing reads by emulating the sequencing process with built-in, technology-specific read error models and base quality value profiles parametrized empirically in large sequencing datasets. Recently, McElroy *et al.* proposed GemSIM [28], creating and using empirically derived, sequence-context based error models to emulate individual sequencing runs technologies, where empirical fragment length and quality score distributions are also used. Wgsim was originally released in the SAMtools software package [29], but in 2011 became a standalone project (https://github.com/lh3/wgsim). Wgsim simulates diploid genomes with SNPs and insertion/deletion (INDEL) polymorphisms and reads with uniform substitution sequencing errors.

In this paper, we describe XS (eXtra Small), a tool for simulation of FASTQ reads, produced by the most known sequencing machines, such as Roche-454, Illumina, ABI SOLiD and, unlike other simulators, Ion Torrent. XS is particularly characterized by flexibility, portability and tunable post-compression ratio. Moreover, it has several running modes depending on the time and memory available, ideal for testing computing infrastructures, namely cloud computing of large-scale projects. XS can be seen as a complementary FASTQ read simulator tool, relative to other methods, since it adds the capacity to change sequence complexity, which naturally can be used in the development and testing of FASTQ compression algorithms.

## Findings

**Figure 1:**
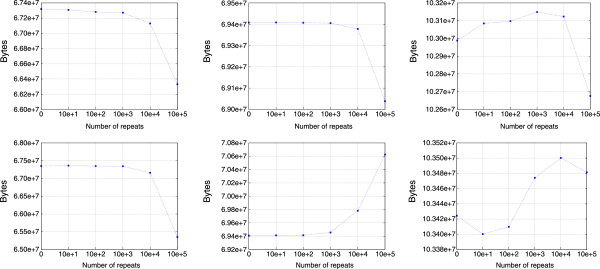
**XS Simulation architecture design.** The architecture of XS simulating a FASTQ file, separating the three different channels of information over the two phases of the simulation process: modeling and generation.

Simulation can be seen as a two stage process: modeling and generation. Modeling is the process of studying the characteristics of the data source to estimate the probability distribution of data. Generation involves the creation of a sequence of symbols according to the probability distribution acquired in the modeling process. Deterministic and stochastic approaches can be adopted in the generation process. XS follows the stochastic approach. Understanding how information is organized in FASTQ files can lead to efficient ways of representing the information (modeling), and hence data simulation. XS simulates FASTQ files with variable size, taking into account the four lines per read basic rules: 

• line 1 begins with a ‘@’ character followed by the header;

• line 2 contains the DNA bases;

• line 3 begins with a ‘+’ character and is optionally followed by the header from line 1;

• line 4 contains the quality scores;

The size of lines 2 and 4 are equal, but can vary from read to read. Since line 3 is an optional repetition of line 1, XS separates the three distinct sources and simulates each one separately, as it can be seen in Figure 1, allowing a more oriented optimization of the simulator for each source, although without forgetting the relations between them, such as the size of line 2, which can be optionally identified in headers, is the same of line 4 and any other base symbol (N) is associated with the lowest quality score. Any of the four lines can be optionally excluded from the file. Therefore, this application can exclude line 1, 3 and 4 and work as a DNA simulator, that can be used to test Transposable Elements (TEs) detection algorithms or DNA substring distribution metrics, such as nucleotide distances [30-33].

### Headers

#### ABI-SOLiD and Illumina

Header formats are so varied and change so frequently that trying to support each one is difficult, even within the same manufacturer. Therefore, XS follows a reasonable line for ABI-SOLiD and Illumina sequencers, generating flow-cells, flow-cell tiles, X and Y coordinates, and pair-ends or the appendices (ex: length=30), according to possible selectable architectures (for each type). Some examples can be seen in the following lines.

ABI-SOLiD examples: 

• @FILEOUT.2286VAB_BARB_20080515_2_Broad_3b_150_1176_133_2030 length=35

• @FILEOUT.2286VAB_solid0019_20100107_QR1004_50x2_QR1000417_21_640/2

Illumina examples: 

• @FILEOUT.2286 HWUSI-EAS100R:1:1:210:308 length=30

• @FILEOUT.2286 HWUSI-EAS100R:6:73:941:1973/1

#### Roche-454

Roche-454 has an alpha-numeric representation based on unique accession numbers with 14 characters, such as the string: C3U5GWL01CBXT2. They consist of 4 components: C3U5GW - a six character encoding of the timestamp of the Run (base-36 encoding); L - a randomizing “hash” character to enhance uniqueness; 01 - indicates the number of regions on the PTP (Pico Titer Plate) which can be between 01 and 16; CBXT2 - a five character encoding of the X, Y location of the well; encoded by computing a total value of “X * 4096 + Y” and encoding that as a five character, base-36 string. To cope with this specification, XS uses the mentioned encoding structure adding at the end of the header the appendix with the respective line size, as can be seen in the following read example. 

• @FILEOUT.2286 C3U5GWL01CBXT2 length=216

#### Ion torrent

Ion Torrent has simpler headers, based on addressable wells (and not on random clusters). These are composed by the run ID (in the following example: QWRK0) and X (01344), Y (01216) coordinates. The maximum value of the coordinates will depend on the chip type (314, 316, 318, P1). For the current largest chip (P1) values are between 0 and 15456 (X coordinate) and 0 and 10656 (Y coordinate). 

• @QWRK0:01344:01216

For all fields of the headers, XS uses a uniform distribution in the stochastic process, mainly because it is fast and also minimizes the probability of repeating fields, when comparing to other distributions.

### DNA bases

Together with the quality-scores, the DNA bases are the most complex types of data. DNA bases, from FASTQ files, are determined by sequences from the 5-symbol alphabet: A (Adenine), C (Cytosine), G (Guanine), T (Thymine), N (any of the previous symbols). These sequences are highly non-stationary and in some regions highly repetitive, usually associated with a particular biological explanation, such as telomers, centromers, transposons, among others [34].

**Figure 2:**
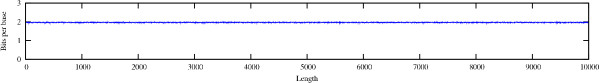
**DNA bases repeats example.** Letter **A** represents an exact copy, **B** represents a reverse complement exact copy and **C** represents an approximate copy. White parts in the main bar represent the unrepeated DNA bases.

**Figure 3:**
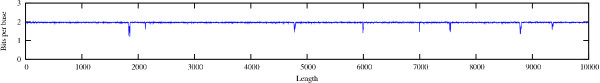
**Information profiles of original and simulated DNA sequences.** The first plot shows 10 Mbp of a regular DNA FASTQ sequence. The rest, also with 10 Mbp, are simulated sequences, respectively, with 0, 10 and 350 repeats, and 350 reverse complemented repeats (repeats minimum size: 1, maximum size: 3000, mutation rate: 0.1). All information profiles have been computed with an encoder based on multiple Markov models and filtered with a window size of 5, from left to right.

Unlike other approaches, XS has two ways to simulate the DNA bases from scratch, without the need for a reference sequence, but maintaining the main characteristics of this type of sequence and therefore improving the portability of the simulator. One is setting both their length and their percentage of nucleotide composition, leading to fast and low memory usage simulation. The other, is an extension of the first, which adds simulation of repeats according to parts of DNA seen in the past. These repeats, exemplified in Figure 2, can be exact or approximate, depending on the average level of mutation selected, ranging between a custom minimum and maximum size. Moreover, there is also the possibility to use the reverse complement repeats, where they can also be exact or approximate copies. An example can be seen in Figure 3, with information profiles assessment [35,36], where the zones of low complexity are related with similarity, and hence, exact or approximate repetitions seen in other parts of the sequence, as in the first plot (real DNA FASTQ sequence). The second plot shows a simulated sequence with absence of repeats, and hence, the zones of low complexity are also absent. In the third, fourth and fifth information profiles, there are regions with low information profiles since the simulator used 10 and 350 repeats. Only the encoder was set to detect reverse complement repeats (fifth information profile) and unset (third and fourth) in order to assess this characteristic of the simulator.

**Figure 4:**
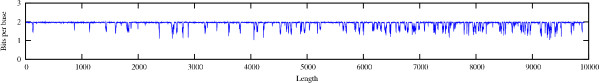
**Compression bytes over two simulations with different settings.** Compression bytes of Fqzcomp (left column), Quip (middle column) and gzip (right column) over several XS simulated FASTQ files with different number of repeats. In the first row, the DNA sequences were simulated without using reverse complemented repeats. In the second row, reverse complemented repeats were used.

As such, the number of repeats used in the simulation increases as the number of compressed bytes values decreases. Figure 4 advocates this matter with Fqzcomp, the winning entry of the Sequence Squeeze competition [37]. Unlike Fqzcomp, Quip is not able to perform well with the increasing of reverse complement repeats, mainly because the model is not prepared to handle this property of the data. Moreover, gzip is unable to perform so well as the other specific methods (Fqzcomp and Quip), which are based on finite-context (Markov) models [38].

The ABI-SOLiD sequencing instruments work on color space and not in sequence space. Thus, XS is prepared to handle this situation using the digits 0 to 3 to encode the color calls (base transitions) according to an equivalence matrix [2].

### Quality scores

Quality scores are numerical values associated with each DNA base in a sequence. These numerical values use a subset of the ASCII printable characters (at most ASCII 33 to 126 inclusive) with a simple offset mapping. However, there are several versions that include different ASCII ranges. Also, there are several lossy compression algorithms using alphabet quantization and recalibration [7,39]. Unlike other approaches, XS interprets variable sparse ASCII ranges. This means, for instance, that it is possible to simulate the following set of ASCII codes: 33, 34, 35, 40–45, 100, 102 and 110–120. In case of score quantization, the post-compression ratio values over the quality scores will decrease as the quantization of the intensities increases, a feature that can be parametrized by the user.

Accordingly, there are two stochastic distributions that can be used in the simulation: uniform and Gaussian. The first, a faster approach, simulates quality scores with equal probability. The second one, simulates quality scores depending on a mean and standard deviation, yielding a simulation that better reflects the nature of biological sequences.

Ion Torrent reads can be very large, resulting both in very long sequences of quality scores and DNA bases, a particularity supported by XS.

## Conclusion

Testing computing infrastructures, namely cloud computing of large-scale projects, and testing FASTQ compression algorithms are some of the examples for which XS can provide simulated data. XS is a FASTQ read simulation tool, characterized by speed, portability, flexibility and with tunable post-compression ratio, that can also be used as a repetitive simulation tool, for example, in Transposable Elements studies. This tool handles Ion Torrent, Roche-454, Illumina and ABI-SOLiD sequencing simulation types, apart from custom handling possibilities.

## Availability and requirements

• **Project name:** XS

• **Project home page:**http://bioinformatics.ua.pt/software/xs/

• **Operating system(s):** Linux

• **Programming language:** C

• **Other requirements:** none

• **License:** GNU GPL v3

• **Any restrictions use by non-academics:** Only those imposed already by the license.

## Competing interests

The authors declare that they have no competing interests.

## Authors’ contributions

DP, AP and JR worked together in the design and testing phase of software development. All authors have read and approved this manuscript.
